# Do Perceptions of Own and Spouse Survival Affect Advance Care Planning?

**DOI:** 10.1093/geroni/igaa057.3522

**Published:** 2020-12-16

**Authors:** Deborah Carr, Yifan Lou

**Affiliations:** 1 Boston University, Boston, Massachusetts, United States; 2 Columbia University, New York, New York, United States

## Abstract

COVID-19 has intensified the need for advance care planning (ACP), or formal preparations for end-of-life care, prior to the time such decisions are required. We propose that older adults’ perceived chances of survival, and one’s perceptions of whether they will outlive their spouse may be powerful motivators of ACP. Using data from the Wisconsin Longitudinal Study (WLS, n=4908, M age = 65), we examine the extent to which: (a) one’s perceived 10- and 20-year survival and (b) projections of dying before, after, or at the same time as one’s spouse affect three aspects of ACP (living will, durable power of attorney for health care designations (DPAHC), and discussions). Multivariate analyses are adjusted for health, demographics, socioeconomic characteristics, and death anxiety. In the full sample, women who perceived a high likelihood of 20-year survival were less likely (OR=.604, p < .05) whereas their male counterparts were more likely (OR = 1.4, p <.01) to name a DPAHC (relative to those who perceived a medium likelihood of survival). Among married persons only (n=3860), people who perceive that they will pre-decease their spouse are 1.5 times as likely to name their spouse as DPAHC (vs. no DPAHC), but are no more likely to name a different person to the role (relative to those who perceive that they and spouse will die at the same time). Practitioner and family conversations about older patients’ projected survival and how it shapes decision making are especially important as COVID-19 may require rapid decisions about end-of-life treatments.

